# Next Generation Sequencing of Genes With Epigenetic Alterations in Mastocytosis

**DOI:** 10.1002/clt2.70106

**Published:** 2025-10-01

**Authors:** Aleksandra Górska, Thierry van De Wetering, Marta Sobalska‐Kwapis, Bogusław Nedoszytko, Danuta Gutowska‐Owsiak, Marek Niedoszytko

**Affiliations:** ^1^ Department of Allergology Medical University of Gdansk Gdansk Poland; ^2^ Department of Genetics and Forensic Genetics Pomeranian Medical University in Szczecin Szczecin Poland; ^3^ Department of Molecular Biophysics University of Lodz Lodz Poland; ^4^ Laboratory of Molecular Basis of Behaviour Nencki Institute of Experimental Biology PAS Warsaw Poland; ^5^ Department of Dermatology Venerology and Allergology Medical University of Gdansk Gdansk Poland; ^6^ Molecular Laboratory Invicta Fertility and Reproductive Centre Sopot Poland; ^7^ Laboratory of Experimental and Translational Immunology Intercollegiate Faculty of Biotechnology University of Gdansk and Medical University of Gdansk University of Gdansk Gdansk Poland

**Keywords:** epigenetics, genes, genetic alterations, mastocytosis, next generation sequencing

## Abstract

**Aim:**

Mastocytosis is a neoplastic disease of the bone marrow associated with the risk of frequent and severe allergic reactions. However, the genetic predisposition is not fully understood, and the crucial element in pathogenesis is the presence of the oncogenic KIT p. D816 V gene mutation. The epigenetic mechanism has also been suggested as playing a role in mastocytosis.

**Objective:**

Based on our previous epigenetic studies, we have selected 110 candidate genes which were sequenced by next generation sequencing (NGS) to identify somatic mutations.

**Method:**

The study group consisted of 32 patients with mastocytosis (16 females and 16 males) plus 16 controls (8 females and 8 males). Whole peripheral blood was collected from all the subjects and genotyped by NGS on the Illumina platform (targeted sequencing).

**Results:**

We analysed 4272 genetic variations in the pre‐selected candidate genes and found five regions that showed a significant difference between the patient and control group. Two of them were found in the *TET2* gene located on chromosome 4 and the other three alterations were found in the genes *DNMT3A*, *SETD2* and *BRD4* located on chromosomes 2, 3 and 19, respectively. Two out of the five genetic variants have not been previously reported, despite the fact that all four genes have been described to be associated with mastocytosis.

**Conclusions:**

The results align with our previous findings, which determined *TET2*, *DNMT3A*, *SETD2* and *BRD4* genes as promising candidates for further analysis, warranting future study in a larger cohort of mastocytosis patients.

## Graphical abstract

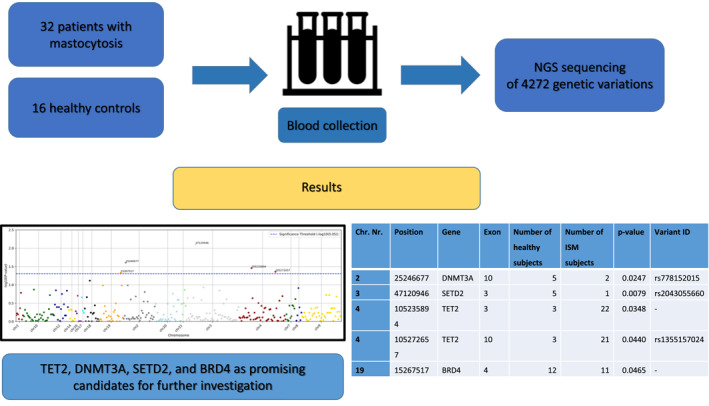

## 1 Introduction

Systemic mastocytosis (SM) is a rare haematological disorder characterized by the expansion and accumulation of atypical mast cells (MCs) in the internal organs, including the bone marrow [1]. Neoplastic MCs are thought to originate from CD34+/CD38‐transformed stem cells [2, 3] which undergo clonal evolution and subclonal expansion as a result of somatic mutations in critical target genes [3]. The clinical course and prognosis of mastocytosis vary significantly based on the WHO‐defined subtype [1]. Patients with cutaneous mastocytosis (CM) or indolent systemic mastocytosis (ISM) generally have a favourable prognosis; ISM, in particular, is typically associated with a normal life expectancy and a minimal risk of progression occurring in less than 5% of patients [1]. In contrast, advanced forms, such as aggressive systemic mastocytosis (ASM) and mast cell leukaemia (MCL), are characterized by rapid progression and poor outcomes [4]. [1].

The crucial element in the pathogenesis of the disease is the presence of the oncogenic *KIT* mutation p.D816 V which is detected in > 80% of all patients with SM and in > 90% of those with ISM [1].

In adult patients with SM these mutations affect mainly the phosphotransferase domain (PTD) of KIT, encoded by exon 17, usually at the position p.816 (i.e., most commonly KIT p.D816 V) [5]. Less frequently, alternative mutations such as p.D816H, p.D816Y, or non‐PTD variants are observed. In rare familial cases, often presenting as paediatric CM, KIT mutations are typically absent or involve uncommon germline variants [5]. SM can be molecularly classified into three subtypes based on KIT mutation status: mast cell–restricted KIT p.D816 V, multilineage KIT p.D816 V, and KIT p.D816 V with additional myeloid‐associated mutations such as TET2, ASXL1, or others [2, 6, 7, 8]. However the presence of the activating *KIT* mutations by themselves are insufficient to explain the varying clinical subtypes of mastocytosis.

Recent studies have identified genetic polymorphisms in cytokine and receptor genes (IL13, IL6, IL6R, IL31, IL4R, VEGFA) and Toll‐like receptors as potential contributors to mastocytosis [8]. The IL4R variant, p.Q576 R, has been associated with CM and improved prognosis [9], while the IL13 promoter variant, −1112C> T, is linked to higher tryptase levels and increased SM risk [10]. Patients with mastocytosis exhibit modified RNA expression profiles in the bone marrow [3]. Transcriptomic analyses of bone marrow in mastocytosis show overexpression of genes involved in mast cell function, including TPSAB1, ATF3, and MAFF, correlating with tryptase levels [11]. Gene expression differences are more pronounced in ASM than ISM, with dysregulation in KIT, CD25, and immune‐related genes (IL1R1, CCL23, CD4). Upregulation of LAT2, CD33, CDH12, and CD81 has also been reported in ISM [12]. Peripheral blood profiling in ISM patients reveals altered expression in pathways, such as JAK‐STAT, MAPK, p53, and ubiquitin‐mediated proteolysis [13]. Although mastocytosis is a distinct clonal mast cell disease, genetic approaches such as Mendelian randomization used in allergic disorders provide an important framework for identifying novel disease‐associated proteins and potential drug targets. For instance, a large Mendelian randomization study demonstrated causal links between plasma protein expression (e.g., TNFAIP3, IL6R, LAYN) and the risk of allergic diseases, suggesting that similar strategies could be applied to uncover therapeutic targets in mastocytosis [14]. Moreover, IL‐33 has been shown to induce CCL2 production in mast cells, promoting basophil migration and modulating endothelial permeability, indicating that comparable pathways may also shape the inflammatory microenvironment in mastocytosis [15]. ISM patients with insect venom–induced anaphylaxis show distinct gene expression profiles compared to those without such reactions [16], while significant expression of *TRAF4* is linked to food hypersensitivity and reduced *B3GAT1* expression is associated with insect venom‐triggered anaphylaxis [17].

While the presence of the oncogenic KIT mutation plays a key role in mastocytosis development, further epigenetic mechanisms might modulate the expression of genes relevant to the pathological process. Recent studies have shown reduced DNA demethylation in the peripheral blood from ISM patients, suggesting an epigenetic contribution to disease pathology. These findings suggest that allergic symptoms in mastocytosis may be associated with DNA demethylation, potentially reflecting an epigenetic activation of immune pathways. In contrast, non‐allergic patients exhibited higher levels of DNA methylation, which may correlate with more profound mast cell dysfunction or a distinct epigenetic profile underlying their clinical presentation [18]. Genome‐wide methylation profiling has identified differentially methylated regions (DMRs) in mastocytosis patients, involving genes related to cellular structure, signal transduction, development, and transcriptional regulation, including KRTCAP3, ANKMY1, GRM2, and the oncogenes FOXQ1, TWIST1, and *ERG* [19]. Summary of genetic, transcriptomic and epigenetic finding associated with mastocytosis is presented in, Supporting Information S1: Table S1.

These findings support the role of epigenetic alterations in mastocytosis initiation and progression, particularly in more advanced disease forms. Therefore, to follow up on our previous findings, we performed targeted NGS of genes identified through epigenetic profiling and assessed their expression using quantitative real‐time PCR (qPCR). This study focuses on the selected candidate genes (Supporting Information S1: Table S2).

## 2 Methods

### 2.1 Study Group

The study comprised 32 ISM patients included in the local registry and treated at the Department of Allergology, Medical University of Gdansk, between 2019 and 2021. Mastocytosis was diagnosed according to the WHO guidelines [20], which included a pathological examination of bone marrow aspirate (cytological evaluation, mast cell immunophenotyping with an assessment of CD2 and CD25 expression), identification of the presence of the activating point mutation in *KIT*, and the serum tryptase level [20]. Molecular detection of the c.2447 A> T variant (KIT p. Asp816Val) in the *KIT* gene was performed using qPCR in bone marrow aspirate. Our local registry is part of the European Competence Network on the Mastocytosis (ECNM) Registry [21]. The control group for the study included 16 adult volunteers, without mastocytosis, matched for sex and age.

## 3 Material Collection

Peripheral blood samples were collected from ISM patients and from healthy volunteers. Informed consent was obtained from all the study participants. The ECNM registry database, data storage, and data distribution conformed to the rules and regulations of the Data Protection Act, the local ethics committee regulations of our centre and the Declaration of Helsinki. Enrolment into the study took place between January 2019 and January 2021. The study was approved by the Independent Bioethics Committee for Scientific Research at the Medical University of Gdańsk: NKBBN/270/2018.

DNA was isolated on the MagNA Pure LC 2.0 instrument using the MagNA Pure LC DNA Isolation Kit I (Roche, Switzerland), according to the manufacturer's protocol. DNA was quantified using a broad range Quant‐iT dsDNA Broad Range Assay Kit (Invitrogen, Carlsbad, CA, USA). Samples with a DNA concentration above 40 ng/μL were included in further analyses if the sex determined from genetic data matched the information provided in the questionnaire. Based on the DNA concentration, the final study group consisted of 32 patients with mastocytosis (16 females and 16 males) and 16 control subjects (8 females and 8 males).

### 3.1 Library Preparation and NGS

**FIGURE 1:**
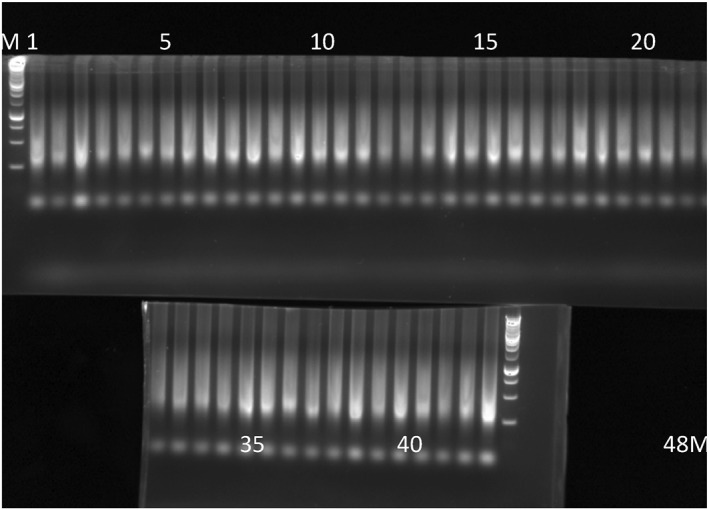
Electrophoretic profiles of prepared DNA libraries.

The Archer VariantPlex HS/HGC Protocol for Illumina (released 12 November 2019) was used to prepare the DNA libraries. A total of 200 ng of DNA per sample was used in a reaction volume of 50 μL. The library preparation consisted of multiple steps. First, DNA was enzymatically fragmented to achieve a size range suitable for sequencing, followed by end repair to create blunt‐ended DNA fragments. In the first ligation step, double‐stranded adaptors containing unique molecular identifiers (UMIs) were added to the DNA ends. These adaptors allowed for accurate variant detection and error correction. Next, molecular redundancy‐controlled (MRC) adaptors were introduced during the second ligation to enable target‐specific enrichment. The library was then amplified using two rounds of polymerase chain reaction (PCR). The first PCR step targeted the ligated products to enrich the adaptor‐ligated DNA fragments, while the second PCR step ensured sufficient yield of the amplified library for sequencing. Cleanup steps were performed after DNA fragmentation, ligation, and PCR amplification to remove excess enzymes, primers, and other reaction by‐products. Cleanup was achieved using paramagnetic beads to purify the DNA fragments and ensure the optimal input for the subsequent steps. To assess the quality and integrity of the DNA fragments, the final libraries were analysed by gel electrophoresis, confirming the expected size distribution (Figure 1).

Based on the above method, 8 samples were combined into 1 pool while for the six different pools the molarity was established with the use of the New England Biolabs: NEBNext Library Quant kit for Illumina on CFX BioRad PCR equipment. The produced results were imported into a dedicated molarity calculator, namely a NEBioCalculator developed by New England Biolabs.

**FIGURE 2:**
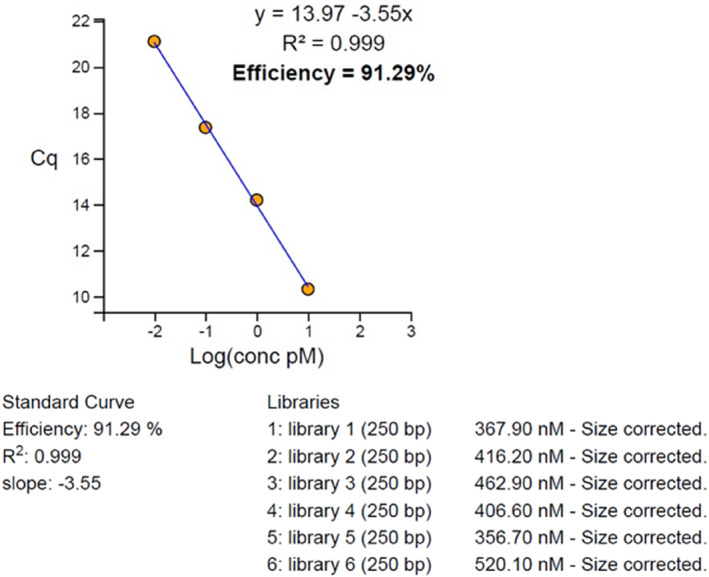
Pooled library concentrations used for final sequencing preparation.

Based on the given values, six pooled libraries were pooled together to create one pool with a final library concentration of 1.6 pM for each sample (Figure 2). The library was loaded into the designated wells of the Illumina MiSeq cartridge, along with the required buffer and flow cell, and subsequently processed using the illumina NextSeq platform.

## 4 Results

The study group consist of 32 ISM patients and 16 adult volunteers, without mastocytosis, matched for sex and age (Table 1).

**TABLE 1:** Baseline clinical characteristics of the study groups.

	Study group, *n* = 32	Control group, *n* = 16
Sex female/male *N*	16/16	8/8
Age range, mean, median	31–66,	34–67,
	49.4,	50,
	49.5	51
Tryptase level mean, median, range	56.7 μg/L,	
	38.8 μg/L,	
	12.5–289 μg/L	
Tryptase level at diagnosis, mean, median, range	45.7 μg/L,	
	35.7 μg/L,	
	10.20–182 μg/L	
Tryptase level at the time of study, mean, median, range	56.7 μg/L,	
	38.8 μg/L,	
	12.5–289 μg/L	
D816 V KIT mutation	Positive 28 (88%),	
	Negative 1 (3%)	
	Not done 2 (6%),	
	No data 1 (3%)	
Mast cell infiltrates in bone marrow biopsy	Positive 21 (66%), Negative 6 (19%),	
	Not done 5 (6%),	
	No data (9%)	
CD2/CD25	Positive 28/28 (87.5%/87.5%)	
	Negative 1/1 (3%/3%)	
	Not done 2/2 (6%/6%)	
	No data 1/1 (3%/3%)	
CD30	Positive 13 (41%)	
	Negative 1 (3%)	
	Not done 17 (53%)	
	No data 1 (3%)	

*Note:* Tryptase level in peripheral blood; KIT mutation in bone marrow aspirate, CD2/CD25 and CD30 in bone marrow aspirate were assessed.

**FIGURE 3:**
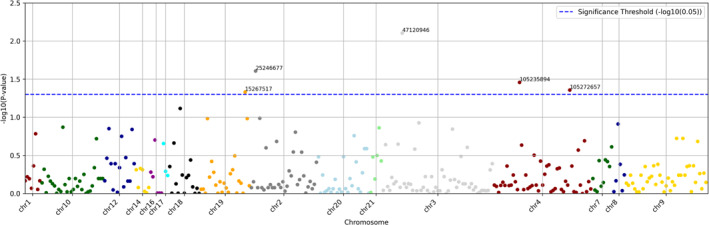
Manhattan Plot depicting significant genetic variants associated with mastocytosis compared to healthy controls.

Initial analysis revealed a cluster density of approximately 318k, which exceeds the optimal range of around 220k for the NextSeq system. The sequencing run generated over 3 million reads, with approximately 84% of the reads successfully identified and mapped to their respective targets. Following the initial assessment, we performed several critical analyses to ensure the accuracy and reliability of our results. This included quality control checks on the read data to filter out low‐quality sequences and remove any duplicates. We also conducted alignment verification using bioinformatics tools to confirm that the reads were accurately mapped to the reference genome. Variant calling identified 4272 genetic variations within the 112 pre‐selected candidate genes, see the Supporting Information S1. Notably, five significant genetic alterations were observed: two in the *TET2* gene on chromosome 4 and three in the *DNMT3A*, *SETD2*, and *BRD4* genes located on chromosomes 2, 3, and 19, respectively. A Manhattan plot analysis was conducted to visualize the significance of these genetic variants across the genome (Figure 3).

Manhattan plot highlighting significant genetic alterations in mastocytosis. Each dot represents a genetic variant, plotted by chromosomal position (*x*‐axis) and –log10‐transformed *p*‐value (*y*‐axis). The blue dashed line indicates the nominal significance threshold (–log10 (0.05)). Five significant variants were identified: two in the TET2 gene on chromosome 4, and one each in DNMT3A (chromosome 2), SETD2 (chromosome 3), and BRD4 (chromosome 19). These findings suggest a potential association between alterations in genes involved in epigenetic regulation and the pathogenesis of mastocytosis. Chromosomes are colour‐coded for clarity.

All four genes have been previously described to be associated with mastocytosis, but two alterations have not been previously reported. Table 2 provides detailed information about the four genes, including their chromosomal location, orientation, and structural features.

**TABLE 2:** Genomic characteristics of detected variants by chromosomal location and gene region annotation.

Gene	Chr.Nr.	Start	Stop	Length on chromosome (bp)	Forward reverse strand	Number of transcripts	Ref Seq	Exons	Coding exons	Transcript length	Amino acids	Full name
SETD2	3	47016429	47164113	147684	Reverse	9	NM_014159.7	21	21	8541	2564	SET domain containing 2, histone lysine methyltransferase (SETD2), transcript variant 1, mRNA
TET2	4	105145875	105279816	133941	Forward	9	NM_001127208.3	11	9	9589	2002	tet methylcytosine dioxygenase 2 (TET2), transcript variant 1, mRNA
DNMT3A	2	25227855	25342590	114735	Reverse	15	NM_022552.5	23	22	9421	912	DNA methyltransferase 3 alpha (DNMT3A), transcript variant 3, mRNA
BRD4	19	15235519	15332539	97020	Reverse	13	NM_001379291.1	20	19	7231	1362	Bromodomain containing 4 (BRD4), transcript variant 4, mRNA

*Note:* This table summarizes the genomic features of each identified variant, including chromosomal location, affected gene, strand orientation, and transcript annotation. Each entry specifies whether the alteration is located in an exon or untranslated region (UTR) and provides details on the corresponding RefSeq transcript, number of coding exons, transcript length, and predicted protein size. Forward/Reverse strand—direction of transcription; Transcript length and amino acids refer to the primary isoform.

Abbreviations: bp: base pairs; Chr.: chromosome; RefSeq: Reference Sequence (NCBI); UTR: untranslated region.

Furthermore, five distinct genetic alterations were significantly associated with disease status when compared to the controls (Table 3). The data includes chromosomal location, associated gene, specific exon, the number of individuals carrying the variant in each group, *p*‐values indicating statistical significance and the variant ID (if available). The findings highlight that the genetic alterations in these genes potentially contribute to the pathogenesis of mastocytosis and warrant further investigation.

**TABLE 3:** Summary of genetic variants with differential occurrence in mastocytosis patients compared to healthy controls.

Chr	Position (bp)	Gene	Exon	Healthy controls (n)	ISM patients (n)	*p*‐value	Variant ID
2	25246677	*DNMT3A*	10	5	2	0.0247	rs778152015
3	47120946	*SETD2*	3	5	1	0.0079	rs2043055660
4	105235894	*TET2*	3	3	22	0.0348	—
4	105272657	*TET2*	10	3	21	0.0440	rs1355157024
19	15267517	*BRD4*	4	12	11	0.0465	—

*Note:* This table lists significant genetic variants identified through comparative analysis between patients with indolent systemic mastocytosis (ISM) and healthy controls. For each variant, the chromosomal location, affected gene and exon, number of individuals with the variant in each group, associated *p*‐value, and corresponding variant are provided.

## 5 Discussion

In this study the NGS analysis of 4272 genetic variants across pre‐selected candidate genes revealed detectable genetic alterations on all chromosomes in peripheral blood cells from both patients and controls. Notably, significant differences between the patient and control groups were observed in the genes DNMT3A, SETD2, TET2, and BRD4, located on chromosomes 2, 3, 4, and 19, respectively. To clarify, DNMT3A is a DNA methyltransferase that regulates gene expression through DNA methylation [22]. It helps restrain mast cell inflammatory responses, and mutations can lead to dysregulated mast cell activation and proliferation. Alterations in this gene might contribute abnormal mast cell behaviour and potentially leading to the disease's progression by altering the mast cell responsiveness to stimuli [23]. We have found a genetic alteration in the gene *DNMT3A* at position 25246677 of chromosome 2 (according to reference genome GRCh38.p14), where 4 nucleotides are deleted (ATTC) and this alteration results in a frameshift mutation. Such frameshift mutation typically results in a premature stop codon, leading to a truncated protein that likely lacks a normal function. This loss‐of‐function mutation can disrupt the normal regulatory mechanisms of mast cell activation and proliferation [23]. Furthermore, different mutations in DNMT3A (including the most prevalent one affecting amino acid R882, as well as frameshift, nonsense, and splice‐site mutations) are predicted to impact translation in patients with mastocytosis [24]. These mutations were significantly enriched in the intermediate‐risk group but were not detected in patients with a favourable‐risk cytogenetic profile in acute myeloid leukaemia [24].

It is important to note that the *SETD2* tumour suppressor gene is responsible for encoding a histone methyltransferase that is involved in both DNA damage repair and translation regulation. It regulates gene expression by modifying DNA methylation patterns [25]. Genetic alterations in the *SETD2* gene may contribute to altered methylation patterns that support mast cell proliferation and survival, potentially influencing mastocytosis development [18]. *SETD2* loss of function was observed in solid and haematologic malignancy. In SM, *SETD2* loss of function occurs mainly at the post‐translational level, rather than at the genomic level, via hyperubiquitination and proteasomal degradation—it is therefore a potentially reversible process that has potential value as a promising therapeutic target for treating patients with AdvSM [25]. We have found a genetic alteration on chromosome 3 at position 47120946 (according to reference genome GRCh38.p14), which occurs to be a silent mutation. Such mutations do not change the amino acid sequence of proteins, but they can still influence gene function by affecting translation efficiency. Kimchi‐Sarfaty et al. have found a silent mutation in the *MDR1* gene which altered the function of P‐glycoprotein, a crucial efflux pump involved in drug resistance, by changing the timing of protein folding during translation [26]. Elongation of the protein folding time may be influenced by the availability of specific transfer RNAs (tRNAs), potentially affecting the final protein conformation and function. Stajic D. et al. indicates that epigenetic gene silencing can drive population adaptation by influencing the mutation rates and types, thereby playing a role in evolutionary processes [27]. This suggests that although silent mutations do not directly change protein sequence, they may affect how genes are expressed or silenced in response to environmental signals.

*TET2* (TET oncogene family member 2) is a candidate tumour suppressor gene located at chromosome 4q24, critical for maintaining the normal haematopoietic function through its role in DNA demethylation [28]. Alterations in this gene may disrupt normal regulatory mechanisms, leading to enhanced mast cell activity and contributing to systemic mastocytosis [29]. In our small study group, we have found two mutations in the *TET2* gene. One of the mutations occurs at position 105272657 (rs1355157024), the alteration changes the amino acid code from Val into Methione or Leucine. The other mutation we have found at position 105235894 of chromosome 4, which is a missense mutation where the amino acid Val is changed into Leu. Such missense mutations can lead to a protein folding change. To our knowledge this mutation has not been described in the literature.

*BRD4*, bromodomain‐containing protein‐4, is involved in regulating transcription and chromatin remodelling. It plays a role in various cellular processes, including inflammation and immune responses. BRD4 interacts with acetylated histones and is also known as an ‘epigenetic reader’ [30]. Although direct links between *BRD4* mutations and mastocytosis are less established, its involvement in transcriptional regulation may influence mast cell behaviour and contribute to disease pathology indirectly. Due to the fact that neoplastic MCs showed significant expression of *BRD4* in ASM and MCL, nowadays *BRD4* has recently emerged as a promising therapeutic target in advanced SM [30].

Over the past decade, genetic influences have been implicated in the pathogenesis of mastocytosis. In particular, the disease development is primarily caused by the somatic KIT p.D816 V mutation, with additional mutations in such genes as TET2, SRSF2, and ASXL1 contributing to advanced disease forms (e.g., ASM, SM‐AHN). Epigenetic dysregulation also plays a role, as it can alter gene expression independently of DNA sequence changes. Our previous research demonstrated reduced 5‐hmC levels in ISM patients and identified 85 differentially methylated regions, including promoter‐associated changes in the oncogenes FOXQ1, TWIST1, and ERG. Additionally, the functional enrichment implicated pathways occur in transcriptional regulation, multicellular development and signal transduction. These results support the routine implementation of advanced technologies, such as droplet digital PCR and NGS into diagnostic and therapeutic strategies for mastocytosis.

## 6 Conclusions

We assume that the currently found mutations highlight the complex interplay between genetic alterations and epigenetic modifications that can drive the pathogenesis of mastocytosis. Moreover, the current results not only support the previous evidence of epigenetic involvement but also highlight TET2, DNMT3A, SETD2, and BRD4 as promising candidates for further investigation. These data warrant validation in larger patient cohorts to better define their diagnostic, prognostic, and therapeutic relevance.

## Author Contributions

**Aleksandra Górska:** conceptualization, investigation, writing – original draft, methodology, writing – review and editing, formal analysis, data curation, resources, visualization. **Thierry van De Wetering:** methodology, validation, visualization, writing – review and editing, formal analysis, data curation. **Marta Sobalska‐Kwapis:** methodology, validation, visualization, formal analysis, data curation. **Bogusław Nedoszytko:** conceptualization, investigation, writing – review and editing, methodology, visualization, supervision, resources. **Danuta Gutowska‐Owsiak:** writing – review and editing, supervision. **Marek Niedoszytko:** conceptualization, investigation, funding acquisition, methodology, validation, visualization, writing – review and editing, formal analysis, supervision, resources, data curation, project administration.

## Conflicts of Interest

The authors declare no conflicts of interest.

## Supporting information


Supporting Information S1

## Data Availability

The authors have nothing to report.
