# Supplementary material for: Next Generation Sequencing of Genes With Epigenetic Alterations in Mastocytosis
Source: Clin Transl Allergy. 2025 Oct 1;15(10):e70106. doi: 10.1002/clt2.70106 (PMC12486320; doi:10.1002/clt2.70106)
Supplement: Supplementary file 1 — Supporting Information S1 [file CLT2-15-e70106-s001.docx]

Next generation sequencing of genes with epigenetic alterations in mastocytotis.

Supplementary file:

Table S1. Summary of genetic, transcriptomic and epigenetic finding associated with mastocytosis.

| Gene | Alteration / Expression Pattern | Associated Condition / Subtype | Biological or Clinical Association |
| --- | --- | --- | --- |
| IL4R | p.Q576R (rs1801275) | Cutaneous Mastocytosis (CM) | Associated with reduced mast cell burden and improved prognosis |
| IL13 | -1112C>T (rs1800925) | Systemic Mastocytosis (SM) | Linked to higher tryptase levels and increased disease risk |
| TPSAB1 | Overexpression | Mastocytosis | Correlates with serum tryptase levels |
| ATF3, MAFF | Overexpression | Mastocytosis | Correlates with mast cell function and tryptase levels |
| KIT, CD25 | Dysregulated expression | Advanced SM (ASM) | More pronounced expression changes than in ISM |
| IL1R1, CCL23, CD4 | Dysregulated expression | ASM > ISM | Reflect immune response disruption |
| LAT2, CD33, CDH12, CD81 | Upregulated | ISM | Involved in degranulation and cell adhesion |
| TRAF4 | Upregulated | ISM with food hypersensitivity | Associated with allergic phenotype |
| B3GAT1 | Downregulated | ISM with insect venom–induced anaphylaxis | Associated with anaphylactic response |
| Various pathways | Expression changes | ISM | Altered JAK-STAT, MAPK, p53, ubiquitin-mediated signalling |
| Global DNA methylation | Decreased demethylation | ISM | Suggests epigenetic involvement in pathogenesis |
| KRTCAP3, ANKMY1, GRM2 | Differentially methylated | Mastocytosis | Linked to cell structure and development |
| FOXQ1, TWIST1, ERG | Differentially methylated oncogenes | Advanced mastocytosis | May contribute to disease progression |

Table S2. The selected candidate genes chosed to assessed in NGS sequencing.

| **GENE** | **Chr.**  **Nr.** | **Start** | **Stop** | **Length on chromosome (bp)** | **Forward / Reverse strand** | **Number of Transcripts** | **RefSeq** | **Exons** | **Coding exons** | **Transcript length** | **Amino acids** | **Full name** |
| --- | --- | --- | --- | --- | --- | --- | --- | --- | --- | --- | --- | --- |
| **ABCA2** | 9 | 137007234 | 137028922 | 21688 | Reverse | 24 | NM_001606.5 | 49 | 49 | 8103 | 2436 | ATP binding cassette subfamily A member 2 (ABCA2), transcript variant 1, mRNA |
| **CDH7** | 18 | 65750252 | 65890337 | 140085 | Forward | 4 | NM_004361.5 | 12 | 11 | 12136 | 785 | cadherin 7 (CDH7), transcript variant b, mRNA |
| **DNMT3A** | 2 | 25227855 | 25342590 | 114735 | Reverse | 15 | NM_022552.5 | 23 | 22 | 9421 | 912 | DNA methyltransferase 3 alpha (DNMT3A), transcript variant 3, mRNA |
| **EAPP** | 14 | 34515938 | 34539711 | 23773 | Reverse | 5 | NM_018453.4 | 6 | 6 | 1303 | 285 | E2F associated phosphoprotein (EAPP), transcript variant 1, mRNA |
| **EDARADD** | 1 | 236348257 | 236502915 | 154658 | Forward | 5 | NM_145861.4 | 6 | 6 | 3088 | 215 | EDAR associated death domain (EDARADD), transcript variant A, mRNA |
| **GRM2** | 3 | 51707068 | 51718613 | 11545 | Forward | 8 | NM_000839.5 | 6 | 5 | 3356 | 872 | glutamate metabotropic receptor 2 (GRM2), transcript variant 1, mRNA |
| **HDAC9** | 7 | 18086949 | 19002416 | 915467 | Forward | 31 | NM_178423.3 | 26 | 25 | 9702 | 1066 | histone deacetylase 9 (HDAC9), transcript variant 4, mRNA |
| **KRTCAP3** | 2 | 27442366 | 27446481 | 4115 | Forward | 7 | NM_173853.4 | 7 | 6 | 872 | 240 | keratinocyte associated protein 3 (KRTCAP3), transcript variant 2, mRNA |
| **OTX2** | 14 | 56799905 | 56816693 | 16788 | Reverse | 11 | NM_021728.4 | 5 | 3 | 3011 | 297 | orthodenticle homeobox 2 (OTX2), transcript variant 1, mRNA |
| **RAB22A** | 20 | 58309715 | 58367507 | 57792 | Forward | 2 | NM_020673.3 | 7 | 7 | 8651 | 194 | RAB22A, member RAS oncogene family (RAB22A), mRNA |
| **RASGEF1B** | 4 | 81426393 | 81471907 | 45514 | Reverse | 14 | NM_152545.3 | 14 | 13 | 2941 | 473 | RasGEF domain family member 1B (RASGEF1B), transcript variant 1, mRNA |
| **RUNX1** | 21 | 34787801 | 36004667 | 1216866 | Reverse | 18 | NM_001754.5 | 9 | 8 | 5971 | 480 | RUNX family transcription factor 1 (RUNX1), transcript variant 1, mRNA |
| **SCG2** | 2 | 223596940 | 223602361 | 5421 | Reverse | 3 | NM_003469.5 | 2 | 1 | 2434 | 617 | secretogranin II (SCG2), mRNA |
| **SGMS1** | 10 | 50305586 | 50625163 | 319577 | Reverse | 11 | NM_147156.4 | 11 | 5 | 3717 | 413 | sphingomyelin synthase 1 (SGMS1), mRNA |
| **SH3PXD2A** | 10 | 103594027 | 103885543 | 291516 | Reverse | 4 | NM_001394015 | 14 | 14 | 11501 | 1105 | SH3 and PX domains 2A (SH3PXD2A), transcript variant 3, mRNA |
| **SLC2A14** | 12 | 7812512 | 7891148 | 78636 | Reverse | 21 | NM_001286234.2 | 11 | 10 | 3462 | 497 | solute carrier family 2 member 14 (SLC2A14), transcript variant 3, mRNA |
| **SETD2** | 3 | 47016429 | 47164113 | 147684 | Reverse | 9 | NM_014159.7 | 21 | 21 | 8541 | 2564 | SET domain containing 2, histone lysine methyltransferase (SETD2), transcript variant 1, mRNA |
| **TET2** | 4 | 105145875 | 105279816 | 133941 | Forward | 9 | NM_001127208.3 | 11 | 9 | 9589 | 2002 | tet methylcytosine dioxygenase 2 (TET2), transcript variant 1, mRNA |
| **TPSAB1** | 16 | 1240379 | 1242554 | 2175 | Forward | 5 | NM_003294.4 | 6 | 5 | 1166 | 275 | tryptase alpha/beta 1 (TPSAB1), mRNA |
| **SLC6A4** | 17 | 30194319 | 30236002 | 41683 | Reverse | 6 | NM_001045.6 | 15 | 13 | 6335 | 630 | solute carrier family 6 member 4 (SLC6A4), mRNA |
| **ASXL1** | 20 | 32358330 | 32439319 | 80989 | Forward | 27 | NM_015338.6 | 13 | 13 | 7052 | 1541 | ASXL transcriptional regulator 1 (ASXL1), transcript variant 1, mRNA |
| **BRD4** | 19 | 15235519 | 15332539 | 97020 | Reverse | 13 | NM_001379291.1 | 20 | 19 | 7231 | 1362 | bromodomain containing 4 (BRD4), transcript variant 4, mRNA |
| **MITF** | 3 | 69739464 | 69968336 | 228872 | Forward | 16 | NM_001354604.2 | 10 | 10 | 4799 | 526 | melanocyte inducing transcription factor (MITF), transcript variant 9, mRNA |
| **MYC** | 8 | 127736231 | 127742951 | 6720 | Forward | 10 | NM_002467.6 | 3 | 3 | 3721 | 454 | MYC proto-oncogene, bHLH transcription factor (MYC), transcript variant 1, mRNA |
| **KIT** | 4 | 54657918 | 54740715 | 82797 | Forward | 4 | NM_000222.3 | 21 | 21 | 5147 | 976 | KIT proto-oncogene, receptor tyrosine kinase (KIT), transcript variant 1, mRNA |
| **IL13** | 5 | 132656263 | 132661110 | 4847 | Forward | 6 | NM_002188.3 | 4 | 4 | 1283 | 146 | interleukin 13 (IL13), transcript variant 1, mRNA |
| **TRAF4** | 17 | 28744005 | 28750956 | 6951 | Forward | 17 | NM_004295.4 | 7 | 7 | 2894 | 470 | TNF receptor associated factor 4 (TRAF4), mRNA |
| **B3GAT1** | 11 | 134378504 | 134412242 | 33738 | Reverse | 5 | NM_054025.3 | 6 | 4 | 3976 | 334 | beta-1,3-glucuronyltransferase 1 (B3GAT1), transcript variant 2, mRNA |
| **CIDEB** | 14 | 24305096 | 24311430 | 6334 | Reverse | 6 | NM_001318807.3 | 8 | 5 | 2409 | 219 | cell death inducing DFFA like effector b (CIDEB), transcript variant 1, mRNA |
| **GADD45A** | 1 | 67685201 | 67688334 | 3133 | Forward | 7 | NM_001924.4 | 4 | 4 | 1352 | 165 | growth arrest and DNA damage inducible alpha (GADD45A), transcript variant 1, mRNA |
| **HRK** | 12 | 116856144 | 116881441 | 25297 | Reverse | 4 | NM_003806.4 | 2 | 1 | 5789 | 91 | harakiri, BCL2 interacting protein (HRK), transcript variant 1, mRNA |
| **TNFRSF25** | 1 | 6460786 | 6466175 | 5389 | Reverse | 21 | NM_003790.3 | 10 | 10 | 1968 | 417 | TNF receptor superfamily member 25 (TNFRSF25), transcript variant 2, mRNA |
| **BIK** | 22 | 43110750 | 43129712 | 18962 | Reverse | 1 | NM_001197.5 | 5 | 4 | 951 | 160 | BCL2 interacting killer (BIK), mRNA |
| **BID** | 22 | 17734138 | 17774770 | 40632 | Reverse | 11 | NM_001196.4 | 6 | 6 | 2177 | 241 | BH3 interacting domain death agonist (BID), transcript variant 2, mRNA |
| **TNFRSF21** | 6 | 47231532 | 47309905 | 78373 | Reverse | 1 | NM_014452.5 | 6 | 6 | 3595 | 655 | TNF receptor superfamily member 21 (TNFRSF21), mRNA |
| **NEUROG1** | 5 | 135534282 | 135535964 | 1682 | Reverse | 1 | NM_006161.3 | 1 | 1 | 1683 | 237 | neurogenin 1 (NEUROG1), mRNA |
| **CDH1** | 16 | 68737292 | 68835537 | 98245 | Forward | 11 | NM_004360.5 | 16 | 16 | 4811 | 882 | cadherin 1 (CDH1), transcript variant 1, mRNA |
| **GSTP1** | 11 | 67583742 | 67586656 | 2914 | Forward | 8 | NM_000852.4 | 7 | 7 | 741 | 210 | glutathione S-transferase pi 1 (GSTP1), mRNA |
| **CDH13** | 16 | 82626969 | 83800640 | 1173671 | Forward | 14 | NM_001257.5 | 14 | 14 | 7876 | 713 | cadherin 13 (CDH13), transcript variant 1, mRNA |
| **TP73** | 1 | 3652516 | 3736201 | 83685 | Forward | 14 | NM_005427.4 | 14 | 13 | 5192 | 636 | tumor protein p73 (TP73), transcript variant 1, mRNA |
| **WIF1** | 12 | 65050626 | 65121305 | 70679 | Reverse | 3 | NM_007191.5 | 10 | 10 | 1977 | 379 | WNT inhibitory factor 1 (WIF1), mRNA |
| **IL4R** | 16 | 27313668 | 27364778 | 51110 | Forward | 19 | NM_000418.4 | 11 | 9 | 3624 | 825 | interleukin 4 receptor (IL4R), transcript variant 1, mRNA |
| **JAK2** | 9 | 4985272 | 5129948 | 144676 | Forward | 4 | NM_004972.4 | 25 | 23 | 7023 | 1132 | Janus kinase 2 (JAK2), transcript variant 1, mRNA |
| **IL9** | 5 | 135892246 | 135895841 | 3595 | Reverse | 1 | NM_000590.2 | 5 | 5 | 605 | 144 | interleukin 9 (IL9), mRNA |
| **RASGRP4** | 19 | 38409051 | 38426305 | 17254 | Reverse | 20 | NM_170604.3 | 17 | 17 | 3132 | 673 | RAS guanyl releasing protein 4 (RASGRP4), transcript variant a, mRNA |
| **ITGB1** | 2 | 9403475 | 9423229 | 19754 | Reverse | 21 | NM_004763.5 | 8 | 6 | 3735 | 200 | integrin subunit beta 1 binding protein 1 (ITGB1BP1), transcript variant 1, mRNA |
| **KMT2C** | 7 | 152134922 | 152436641 | 301719 | Reverse | 28 | NM_170606.3 | 59 | 59 | 16860 | 4911 | lysine methyltransferase 2C (KMT2C), mRNA |
| **ITGAV** | 2 | 186590056 | 186680901 | 90845 | Forward | 8 | NM_002210.5 | 30 | 30 | 7039 | 1048 | integrin subunit alpha V (ITGAV), transcript variant 1, mRNA |
| **IL6** | 7 | 22725884 | 22732002 | 6118 | Forward | 9 | NM_000600.5 | 6 | 5 | 1127 | 212 | interleukin 6 (IL6), transcript variant 1, mRNA |
| **IL6R** | 1 | 154405193 | 154469450 | 64257 | Forward | 8 | NM_000565.4 | 10 | 10 | 5764 | 468 | interleukin 6 receptor (IL6R), transcript variant 1, mRNA |
| **IL-31** | 12 | 122172029 | 122174221 | 2192 | Reverse | 1 | NM_001014336.2 | 3 | 3 | 927 | 164 | interleukin 31 (IL31), mRNA |
| **VEGFA** | 6 | 43770184 | 43786487 | 16303 | Forward | 26 | NM_003376.6 | 7 | 7 | 3535 | 371 | vascular endothelial growth factor A (VEGFA), transcript variant 2, mRNA |
| **TLR2** | 4 | 153684070 | 153706260 | 22190 | Forward | 8 | NM_001318789.2 | 3 | 1 | 3596 | 784 | toll like receptor 2 (TLR2), transcript variant 2, mRNA |
| **TLR4** | 9 | 117704175 | 117724735 | 20560 | Forward | 4 | NM_138554.5 | 3 | 3 | 12677 | 839 | toll like receptor 4 (TLR4), transcript variant 1, mRNA |
| **TLR9** | 3 | 52221080 | 52225645 | 4565 | Reverse | 1 | NM_017442.4 | 2 | 2 | 3352 | 1032 | toll like receptor 9 (TLR9), mRNA |
| **RAB27A** | 15 | 55202966 | 55319113 | 116147 | Reverse | 12 | NM_183235.3 | 6 | 5 | 3447 | 221 | RAB27A, member RAS oncogene family (RAB27A), transcript variant 3, mRNA |
| **ETS1** | 11 | 128458761 | 128587558 | 128797 | Reverse | 9 | NM_001143820.2 | 10 | 9 | 5139 | 485 | ETS proto-oncogene 1, transcription factor (ETS1), transcript variant 1, mRNA |
| **OR51Q1** | 11 | 5422111 | 5423206 | 1095 | Forward | 1 | NM_001004757.2 | 1 | 1 | 1096 | 317 | olfactory receptor family 51 subfamily Q member 1 (OR51Q1), mRNA |
| **CYP2B6** | 19 | 40991282 | 41018398 | 27116 | Forward | 5 | NM_000767.5 | 9 | 9 | 3071 | 491 | cytochrome P450 family 2 subfamily B member 6 (CYP2B6), mRNA |
| **RPTN** | 1 | 152153595 | 152159228 | 5633 | Reverse | 1 | NM_001122965.1 | 3 | 2 | 3569 | 784 | repetin (RPTN), mRNA |
| **PDE4DIP** | 1 | 148808181 | 149048286 | 240105 | Forward | 50 | NM_001350520.1 | 44 | 44 | 8307 | 2362 | phosphodiesterase 4D interacting protein (PDE4DIP), transcript variant 10, mRNA |
| **FTCD** | 21 | 46136262 | 46155579 | 19317 | Reverse | 12 | NM_206965.2 | 15 | 15 | 541 | 572 | formimidoyltransferase cyclodeaminase (FTCD), transcript variant A, mRNA |
| **ZNF134** | 19 | 57614233 | 57624724 | 10491 | Forward | 4 | NM_003435.5 | 3 | 2 | 4924 | 427 | zinc finger protein 134 (ZNF134), mRNA |
| **FXR1** | 3 | 180912670 | 180982753 | 70083 | Forward | 22 | NM_005087.4 | 17 | 17 | 8343 | 621 | FMR1 autosomal homolog 1 (FXR1), transcript variant 1, mRNA |
| **MSH5** | 6 | 31739677 | 31762676 | 22999 | Forward | 21 | NM_172166.4 | 25 | 24 | 2721 | 834 | mutS homolog 5 (MSH5), transcript variant 4, mRNA |
| **MOCS1** | 6 | 39899578 | 39934551 | 34973 | Reverse | 9 | NM_001358530.2 | 11 | 11 | 4143 | 636 | molybdenum cofactor synthesis 1 (MOCS1), transcript variant 7, mRNA |
| **CSNK1E** | 22 | 38290691 | 38318084 | 27393 | Reverse | 15 | NM_152221.3 | 11 | 9 | 2817 | 416 | casein kinase 1 epsilon (CSNK1E), transcript variant 1, mRNA |
| **AC007682.1** | 2 | 51011777 | 51016950 | 5173 | Forward | 1 | AC007682.1 | 3 | 0 | 1852 | 0 | BAC clone RP11-391D19 from 2, complete sequence |
| **ARHGAP42** | 11 | 100687288 | 100993941 | 306653 | Forward | 9 | NM_152432.4 | 24 | 24 | 8156 | 874 | Rho GTPase activating protein 42 (ARHGAP42), transcript variant 1, mRNA |
| **S100A7A** | 1 | 153416520 | 153423222 | 6702 | Forward | 3 | NM_176823.4 | 3 | 2 | 4280 | 101 | S100 calcium binding protein A7A (S100A7A), mRNA |
| **TRPV6** | 7 | 142871208 | 142885745 | 14537 | Reverse | 8 | NM_018646.6 | 15 | 15 | 2906 | 765 | transient receptor potential cation channel subfamily V member 6 (TRPV6), mRNA |
| **DYNC2I1** | 7 | 158856558 | 158956747 | 100189 | Forward | 5 | NM_018051.5 | 25 | 25 | 3789 | 1066 | dynein 2 intermediate chain 1 (DYNC2I1), transcript variant 1, mRNA |
| **POLQ** | 3 | 121431427 | 121546641 | 115214 | Reverse | 4 | NM_199420.4 | 30 | 30 | 8757 | 2590 | DNA polymerase theta (POLQ), mRNA |
| **LINC00381** | 13 | 74419158 | 74444735 | 25577 | Forward | 2 | NR_047005.1 | 5 | 0 | 1280 | 0 | LINC00381 long intergenic non-protein coding RNA 381 [ (human) ] |
| **LINC01412** | 2 | 144523912 | 144579434 | 55522 | Forward | 1 |  | 3 | 0 | 598 | 0 | LINC01412 long intergenic non-protein coding RNA 1412 [ (human) ] |
| **TBL1XR1** | 3 | 177019340 | 177228000 | 208660 | Reverse | 36 | NM_024665.7 | 16 | 14 | 8182 | 514 | TBL1X receptor 1 (TBL1XR1), transcript variant 1, mRNA |
| **TERT** | 5 | 1253147 | 1295068 | 41921 | Reverse | 7 | NM_198253.3 | 16 | 16 | 4039 | 1132 | telomerase reverse transcriptase (TERT), transcript variant 1, mRNA |
| **BCR** | 22 | 23179704 | 23318037 | 138333 | Forward | 15 | NM_004327.4 | 23 | 23 | 6783 | 1271 | BCR activator of RhoGEF and GTPase (BCR), transcript variant 1, mRNA |
| **ABL1** | 9 | 130713016 | 130887675 | 174659 | Forward | 3 | NM_005157.6 | 11 | 11 | 5578 | 1130 | ABL proto-oncogene 1, non-receptor tyrosine kinase (ABL1), transcript variant a, mRNA |
| **TEX41** | 2 | 144667985 | 145076729 | 408744 | Forward | 189 | NR_033870.2 | 4 | 0 | 4635 | 0 | TEX41 testis expressed 41 [ (human) ] |
| **ZEB2** | 2 | 144364364 | 144521057 | 156693 | Reverse | 49 | NM_014795.4 | 10 | 9 | 9265 | 1214 | zinc finger E-box binding homeobox 2 (ZEB2), transcript variant 1, mRNA |
| **CHEK2** | 22 | 28687743 | 28742422 | 54679 | Reverse | 25 | NM_007194.4 | 16 | 15 | 1844 | 586 | checkpoint kinase 2 (CHEK2), transcript variant 1, mRNA |
| **GFI1B** | 9 | 132944000 | 132991687 | 47687 | Forward | 8 | NM_001377304.1 | 11 | 6 | 1798 | 330 | growth factor independent 1B transcriptional repressor (GFI1B), transcript variant 4, mRNA |
| **ZNF521** | 18 | 25061924 | 25352190 | 290266 | Reverse | 14 | NM_015461.3 | 8 | 7 | 4887 | 1311 | zinc finger protein 521 (ZNF521), transcript variant 1, mRNA |
| **GATA2** | 3 | 128479427 | 128493201 | 13774 | Reverse | 6 | NM_032638.5 | 6 | 5 | 3383 | 480 | GATA binding protein 2 (GATA2), transcript variant 2, mRNA |
| **MECOM** | 3 | 169083499 | 169663775 | 580276 | Reverse | 20 | NM_004991.4 | 17 | 17 | 5462 | 1239 | MDS1 and EVI1 complex locus (MECOM), transcript variant 4, mRNA |
| **HMGA1** | 6 | 34236873 | 34246231 | 9358 | Forward | 7 | NM_145899.3 | 5 | 4 | 1920 | 107 | high mobility group AT-hook 1 (HMGA1), transcript variant 1, mRNA |
| **SLC12A7** | 5 | 1050384 | 1112063 | 61679 | Reverse | 6 | NM_006598.3 | 24 | 24 | 5300 | 1083 | solute carrier family 12 member 7 (SLC12A7), mRNA |
| **SH2B3** | 12 | 111405923 | 111451623 | 45700 | Forward | 3 | NM_005475.3 | 8 | 7 | 5431 | 575 | SH2B adaptor protein 3 (SH2B3), transcript variant 1, mRNA |
| **SMC4** | 3 | 160399274 | 160434954 | 35680 | Forward | 27 | NM_001002800.3 | 23 | 23 | 5116 | 1288 | structural maintenance of chromosomes 4 (SMC4), transcript variant 2, mRNA |
| **MAD1L1** | 7 | 1815793 | 2233243 | 417450 | Reverse | 23 | NM_001013836.2 | 19 | 17 | 2695 | 718 | mitotic arrest deficient 1 like 1 (MAD1L1), transcript variant 2, mRNA |
| **ATM** | 11 | 108222832 | 108369102 | 146270 | Forward | 31 | NM_000051.4 | 63 | 62 | 12915 | 3056 | ATM serine/threonine kinase (ATM), transcript variant 2, mRNA |
| **FOXO1** | 13 | 40555667 | 40666641 | 110974 | Reverse | 4 | NM_002015.4 | 3 | 2 | 5779 | 655 | forkhead box O1 (FOXO1), mRNA |
| **CD44** | 11 | 35138882 | 35232402 | 93520 | Forward | 39 | NM_000610.4 | 18 | 18 | 5431 | 742 | CD44 molecule (Indian blood group) (CD44), transcript variant 1, mRNA |
| **KRAS** | 12 | 25205246 | 25250936 | 45690 | Reverse | 4 | NM_004985.5 | 5 | 4 | 5306 | 188 | KRAS proto-oncogene, GTPase (KRAS), transcript variant b, mRNA |
| **NRAS** | 1 | 114704469 | 114716771 | 12302 | Reverse | 1 | NM_002524.5 | 7 | 4 | 4326 | 189 | NRAS proto-oncogene, GTPase (NRAS), mRNA |
| **HRAS** | 11 | 532242 | 537287 | 5045 | Reverse | 11 | NM_005343.4 | 6 | 4 | 1070 | 189 | HRas proto-oncogene, GTPase (HRAS), transcript variant 1, mRNA |
| **PDGFRB** | 5 | 150113839 | 150155872 | 42033 | Reverse | 11 | NM_002609.4 | 23 | 22 | 5700 | 1106 | platelet derived growth factor receptor beta (PDGFRB), transcript variant 1, mRNA |
| **STAT5A** | 17 | 42287547 | 42311943 | 24396 | Forward | 15 | NM_001288718.2 | 19 | 18 | 3770 | 794 | signal transducer and activator of transcription 5A (STAT5A), transcript variant 1, mRNA |
| **STAT5B** | 17 | 42199177 | 42276707 | 77530 | Reverse | 7 | NM_012448.4 | 19 | 18 | 5079 | 787 | signal transducer and activator of transcription 5B (STAT5B), mRNA |
| **PIM1** | 6 | 37170152 | 37175428 | 5276 | Forward | 3 | NM_002648.4 | 6 | 6 | 2703 | 313 | Pim-1 proto-oncogene, serine/threonine kinase (PIM1), transcript variant 1, mRNA |
| **BCL2** | 18 | 63123346 | 63320128 | 196782 | Reverse | 9 | NM_000633.3 | 3 | 2 | 6881 | 239 | BCL2 apoptosis regulator (BCL2), transcript variant alpha, mRNA |
| **MCL1** | 1 | 150560895 | 150579738 | 18843 | Reverse | 8 | NM_021960.5 | 3 | 3 | 3950 | 350 | MCL1 apoptosis regulator, BCL2 family member (MCL1), transcript variant 1, mRNA |
| **BCL2L11** | 2 | 111119378 | 111168445 | 49067 | Forward | 23 | NM_138621.5 | 4 | 3 | 5098 | 198 | BCL2 like 11 (BCL2L11), transcript variant 1, mRNA |
| **BBC3** | 19 | 47220822 | 47232766 | 11944 | Reverse | 6 | NM_014417.5 | 4 | 3 | 1846 | 193 | BCL2 binding component 3 (BBC3), transcript variant 4, mRNA |
| **BAX** | 19 | 48954875 | 48961798 | 6923 | Forward | 13 | NM_138761.4 | 6 | 6 | 795 | 192 | BCL2 associated X, apoptosis regulator (BAX), transcript variant alpha, mRNA |
| **NOXA** | 9 | 137423350 | 137434406 | 11056 | Forward | 2 | NM_001256067.2 | 14 | 14 | 1678 | 483 | NADPH oxidase activator 1 isoform 2 |
| **HMOX1** | 22 | 35380361 | 35394214 | 13853 | Forward | 7 | NM_002133.3 | 5 | 5 | 1554 | 288 | heme oxygenase 1 (HMOX1), mRNA |
| **HSPBP1** | 19 | 55262223 | 55280110 | 17887 | Reverse | 9 | NM_012267.5 | 8 | 7 | 1768 | 359 | HSPA (Hsp70) binding protein 1 (HSPBP1), transcript variant 1, mRNA |
| **HSP90AA1** | 14 | 102080742 | 102139699 | 58957 | Reverse | 10 | NM_005348.4 | 11 | 10 | 3228 | 732 | heat shock protein 90 alpha family class A member 1 (HSP90AA1), transcript variant 2, mRNA |
